# Transcriptome of dorsal root ganglia caudal to a spinal cord injury with modulated behavioral activity

**DOI:** 10.1038/s41597-019-0088-4

**Published:** 2019-06-07

**Authors:** Julia H. Chariker, Cynthia Gomes, Fiona Brabazon, Kathryn A. Harman, Sujata Saraswat Ohri, David S. K. Magnuson, Scott R. Whittemore, Jeffrey C. Petruska, Eric C. Rouchka

**Affiliations:** 1 Department of Neuroscience Training, University of Louisville, 522 East Gray Street, Louisville, Kentucky 40202 USA; 2 Kentucky Biomedical Research Infrastructure Network Bioinformatics Core, 522 East Gray Street, Louisville, Kentucky 40202 USA; 3 Department of Anatomical Sciences and Neurobiology, University of Louisville, 511 South Floyd St., Louisville, KY 40202 USA; 4 Kentucky Spinal Cord Injury Research Center, University of Louisville, 511 South Floyd St., Louisville, KY 40202 USA; 5 Department of Neurological Surgery, University of Louisville, 220 Abraham Flexner Way, Suite 1500, Louisville, KY 40202 USA; 6 Department of Health & Sport Sciences, University of Louisville, 2100 South Floyd Street, Louisville, KY 40208 USA; 7 Department of Computer Engineering and Computer Science, Speed School of Engineering, University of Louisville, Duthie Center for Engineering, 2301 South 3rd St., Louisville, Kentucky 40292 USA; 8 Present Address: Wiley Publishing, Hoboken, NJ 07030 USA

**Keywords:** Spinal cord injury, High-throughput screening, Transcriptomics

## Abstract

Spinal cord injury (SCI) is a devastating clinical condition resulting in significant disabilities. Apart from local injury within the spinal cord, SCI patients develop a myriad of complications including multi-organ dysfunction. Some of the dysfunctions may be directly or indirectly related to the sensory neurons of the dorsal root ganglia (DRG), which signal to both the spinal cord and the peripheral organs. After SCI, some classes of DRG neurons exhibit sensitization and undergo axonal sprouting both peripherally and centrally. Such physiological and anatomical re-organization after SCI contributes to both adaptive and maladaptive plasticity processes, which may be modulated by activity and exercise. In this study, we collected comprehensive gene expression data in whole DRG below the levels of the injury to compare the effects of SCI with and without two different forms of exercise in rats.

## Abstract (summary)

**Table:** 

Design Type(s)	gene expression analysis objective • stimulus or stress design • transcription profiling design
Measurement Type(s)	gene expression
Technology Type(s)	RNA sequencing
Factor Type(s)	Spinal Cord Injury • Exercise • biological replicate
Sample Characteristic(s)	Rattus norvegicus • dorsal root ganglion

Machine-accessible metadata file describing the reported data (ISA-Tab format)

## Background & Summary

Injury to the spinal cord leads to many complications, ranging from the more observable such as loss of motor control, to the less observable, such as multi-organ dysfunction, chronic pain, and autonomic dysregulation. With reduced or absent descending control (pathways originating in the brain that project onto the spinal cord^1^, leading to what is known as supraspinal input) following SCI, spinal circuitry becomes highly reliant on information coming from the periphery and carried by the sensory neurons^2^. This makes understanding the response of sensory neurons to SCI and treatments of paramount importance. A growing body of work indicates that although the vast majority of spinal sensory neurons housed in DRG do not appear to be directly injured by SCI, alterations in sensory neuron anatomy and physiology not only contribute to life-threatening conditions that develop after SCI but also contribute to some forms of recovered functions. Below the level of injury, some sensory neurons undergo intra-spinal axonal sprouting which provides new input to spinal circuitry^3,4^. In one example, sprouting of nociceptive axons with concomitant SCI-induced loss of descending controls allows sensory neurons unchecked access to sensory, autonomic, and motor circuitry, which contributes to chronic pain, autonomic dysreflexia, and impaired locomotion^5–7^. However, such plasticity can also be beneficial in enhancing outcomes of locomotor training, where inputs from skin and muscle are major drivers^8–10^. In addition to inducing intra-spinal axonal sprouting, SCI also leads to persistent hyperexcitability and spontaneous activity in some neuronal cell bodies and peripheral branches of DRG neurons^11^, which could be a major contributor to developing chronic pain and autonomic dysregulation.

Previous studies have shown spontaneous improvement in locomotor function following sufficient “in-cage activity” after SCI^12–15^. Therefore, as a control, this study analyzed DRG from rats housed in locomotor-restricting (tiny) cages where in-cage activity was reduced by 75–80% for both SCI and naïve groups (DSKM, unpublished observations). This type of housing mimics the clinical situation in which patients are bed- and wheelchair- bound for weeks or months following injury. Spinal cord injured rats, all of which were housed in tiny cages, were divided into two injury groups: contusion SCI (CONT SCI), which resulted in some sparing of supraspinal input, and complete spinal cord transection (TX SCI), which resulted in the total loss of descending, supraspinal input. In addition, subsets of CONT SCI animals were randomly assigned to two exercise training paradigms: swimming (SWIM) and shallow water walking (SWW). These models of enhanced activity/exercise training have beneficial effects in terms of recovery of function and complement our previous study of transcriptional responses in liver^14–18^. Further, several clinical and pre-clinical studies suggest that physical activity and exercise can be effective in enhancing recovery of function and attenuating SCI-induced neuropathic pain and autonomic dysregulation in both humans and rodents^7,19–22^. Exercise training ameliorates chronic pain in rodents and is associated with reducing SCI-induced intra-spinal axonal sprouting of subsets of afferents^7,20^. Exercise can also influence sensory neuron function and transcription in intact, nerve-injured, and SCI conditions^7,23,24^. Intrinsic changes in gene expression could underlie SCI-induced neuronal plasticity (sprouting and sensitization) in DRG neurons and the prevention/reversal with exercise.

These data were collected to evaluate the transcriptomic changes in DRG below the level of SCI and to better understand how this profile is altered with exercise training. Because the study was designed to identify broad effects at the transcriptional level, as opposed to organ/tissue/segment-specific effects, bilateral DRG from many segments below the injury were pooled within animals. Our technical validation indicates that the data are of high quality with gene expression occurring in expected gene regions. UCSC Genome Browser expression tracks are available with the raw data to facilitate exploration of the samples. The data described in this paper are offered to the scientific community as a valuable resource for future investigations into the molecular changes in DRG occurring in SCI-induced pathologies.

## Methods

### Animals

All animal procedures were performed in accordance with the Public Health Service Policy on Humane Care and Use of Laboratory Animals (Institute of Laboratory Animal Resources, National Research Council, 1996) and the University of Louisville Institutional Animal Care and Use Committee. Female Sprague Dawley rats of body weight 235–249 g (~8 to 9 weeks old) were obtained from Sprague Dawley, Inc. (Indianapolis, IN). We chose to work exclusively with female adult rats to control for transcriptomic responses related to age and gender. Also, in our experience, female rats recover more quickly from surgery and are more motivated to exercise, providing better outcome measures for our experimental purposes. All rats were housed initially in standard cages and maintained in a continuous 12 h-light/dark cycle. Tap water and a standard rodent diet were available to all rats *ad libitum*.

### Experimental design and SCI

**Fig. 1:**
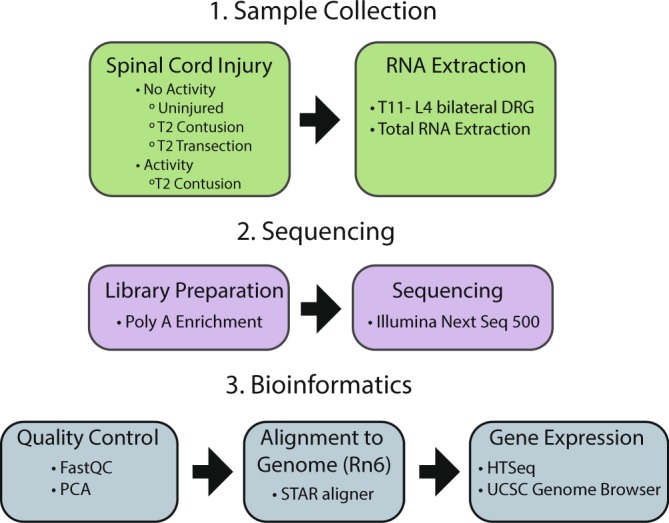
Experimental workflow. (1) Rats received a contusion SCI, a transection SCI, or no SCI. A subset of rats with contusion SCI were exposed to swimming or shallow water walking exercise training after injury. RNA was extracted from both the left and right dorsal root ganglia (DRG) at T11 through L4. (2) RNA samples were subjected to poly A enrichment and sequenced on an Illumina NextSeq 500. (3) The bioinformatics workflow included a quality control analysis, alignment to the *Rattus norvegicus* (Rn6) reference assembly, and gene expression analysis with UCSC Genome Browser visualization.

**Fig. 2:**
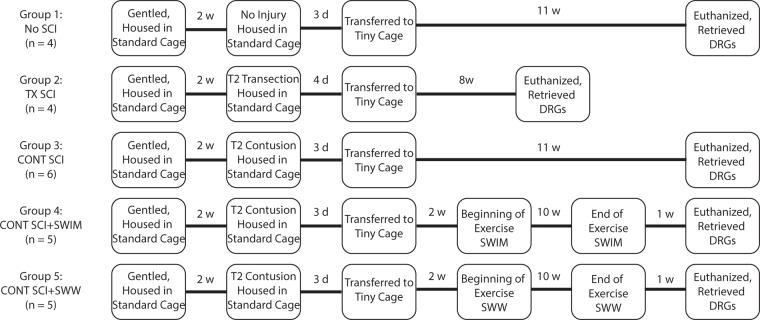
Experimental Paradigm. Rats were gentled for two weeks. After two weeks, a moderate-severe T2 contusion (Groups 3, 4 and 5) or complete T2 transection (Group 2) was performed. After 3~4 days, rats in all groups were transferred to tiny cages. Two weeks post-transfer, a subset of rats with contusion SCI were given swimming or shallow water walking exercise training for 10 weeks (5 days per week). All groups were euthanized after designated time points for terminal assessments.

The experimental workflow is displayed in Fig. 1 with the experimental timeline for SCI with and without exercise, given in greater detail in Fig. 2. Prior to the study, 24 animals were randomly assigned to five groups: naïve (No SCI, 4 replicates), T2 transection injury (TX SCI, 4 replicates), T2 contusion injury (CONT SCI, 6 replicates), T2 contusion injury followed by swimming as exercise (CONT SCI + SWIM, 5 replicates), T2 contusion injury followed by shallow water walking as exercise (CONT SCI + SWW, 5 replicates). Throughout the study, rats were doubly-housed with animals in the same experimental group.

All rats were initially gentled and acclimatized to testing and exercise facilities for two weeks. After this period, animals were anaesthetized with ketamine (50 mg/kg)/xylazine (0.024 mg/kg)/acepromazine (0.005 mg/kg) cocktail (IP) and glycopyrolate (0.08 mg/kg, IM) was given prior to SCI surgeries. For all injury groups (both TX SCI and CONT SCI), a dorsal midline incision was made in the superficial muscle overlying the T1-T3 vertebrae. A single level laminectomy was performed on the T2 vertebra; dura mater was kept intact. For animals in the TX SCI group, complete transection of the spinal cord at T2 was performed with a scalpel. Animals in the CONT SCI group received a moderately-severe contusion injury (25 g-cm) at the T2 spinal cord level using the NYU Impactor^25,26^. Injured animals were monitored on heating pads until they recovered from the anaesthesia. Rats were then double housed in standard cages with ALPHA-dri® bedding (Shepherd’s^TM^ Specialty Paper, Milford, NJ). Post operative care consisted of prophylactic antibiotics (gentamicin sulphate, 20 mg/kg, SC), given for 7 consecutive days. For pain management injured animals received buprenorphine (0.03 mg/kg, SC) twice daily for three days and as needed thereafter. Animals were also given 5 ml boluses of lactated Ringer’s solution for three days (and as needed for hydration thereafter). Manual bladder expression was performed thrice daily for each animal until reflexive voiding was re-established.

Three days after injury, all animals (naïve included) were doubly-housed with animals in the same experimental group in tiny cages (7.5′′ × 8.5′′ × 8′′) rather than standard cages (22′′ × 12.5′′ × 8′′) to restrict their overground locomotion for the duration of the study. If mortality resulted in an odd number of animals, then an injured animal not in the study was used as a cage mate. For animals in the exercise training groups, swimming or shallow water walking began 2.5 weeks post-injury and was conducted 5 consecutive days a week for 10 weeks. Animals exercised for a total of 30 minutes each day with 15 minutes of exercise in the morning and 15 minutes in the afternoon. The morning and afternoon sessions were separated by a minimum of one hour. Each 15 minute session consisted of three 5 minute periods of exercise with breaks between the periods lasting approximately 20–25 minutes.

### Tissue collection and RNA extraction

Animals were sacrificed with a ketamine overdose at 8.5, 11.5 or 13.5 weeks post-surgery depending on experimental condition (see Fig. 2). Hearts were arrested in diastole with an injection of 3 M KCl. Animals were perfused with PBS supplemented with 20% RNA*later* (Thermo Fisher Scientific, Waltham, MA). For each animal, T11-L4 DRG were retrieved from both sides of the spinal column and collected together in QIAzol (Qiagen, Germantown, MD). Total RNA from each sample was isolated with RNeasy Lipid Tissue Mini Kit (Qiagen).

### Library preparation and sequencing

1 ug of total RNA samples were used for poly A enrichment. First and second strands were synthesised followed by 3′ end adenylation. Samples were barcoded with Illumina TrueSeq adapters. 1.8 pM of barcoded library was denatured, and sequencing was performed on the University of Louisville Center for Genetics and Molecular Medicine (CGeMM) Illumina NextSeq 500 using the NextSeq 500/550 1X75 cycle high output kit.

### RNA-seq data analysis

Sequencing produced nearly a billion single end reads across the 24 samples. The vast majority of read lengths fell between 74 and 76 bases across all samples. The quality of the reads was assessed using FastQC v.0.10.1^27^, which indicated no sequence trimming was necessary. The sequences were directly aligned to the *Rattus norvegicus* reference genome assembly (Rn6) using the STAR aligner v.2.6^28^. Read counts were extracted with HTSeq version 0.10.0^29^, using Rn6 Ensembl annotations (build 93) to locate gene regions^30^. Prior to extracting read counts, mitochondrial genes were removed from the annotation file to reduce non-relevant variation in future analyses. This produced an annotation file testing 24,613 regions. Prior to examining expression, raw read counts were normalized using DESeq2’s default method, relative log expression (RLE)^31,32^. UCSC Genome Browser tracks were created to easily explore expression across the tested sites^33^. The tracks were created using guidelines and utilities available on the UCSC Genome Browser website. Much of this involved converting sequencing alignment files in BAM format to BigWig format, required for use on the browser.

A principal component analysis (PCA) was performed using the plotPCA function in DESeq2. Prior to the analysis, a variance stabilizing transformation was performed on the raw read count matrix to achieve similar variance at all levels of mean expression across the samples. This minimizes the effect of highly varying genes on the spread of points in the PCA plot. The PCA plot was generated using the R programming language package ggplot2^34^.

## Data Records

The data were submitted to NCBI Gene Expression Omnibus (GSE125630)^35^. This GEO project includes raw data in Fastq format, raw HTSeq^29^ counts, and UCSC Genome Browser^33^ tracks in bigwig format for all samples. This dataset is part of a larger study measuring the systemic transcriptional response to spinal cord injury, including liver^16^ and soleus muscle, all of which are included as part of a GEO superseries (GSE129704)^36^.

## Technical Validation

*RNA metrics:* Sequencing generated 22.8 to 54.7 million reads/sample with a mean of 40.7 million and standard deviation of 8 million. Table 1 displays the number of raw reads successfully aligned for each of the samples. The alignment rate for uniquely mapped and multi-mapped reads combined ranged from 98 to 98.6 percent with a mean of 98.4 across the 24 samples.

**Table 1:** Sequencing and alignment summary.

Sample ID	Experimental Group	Input Reads	Number Uniquely Mapped Reads	Percent Uniquely Mapped Reads	Number Multi-mapped Reads	Percent Multi-mapped Reads
No SCI, Replicate 1	No SCI	43,326,516	38,768,226	89.48%	3,919,395	9.05%
No SCI, Replicate 2	No SCI	42,009,023	37,683,145	89.70%	3,696,445	8.80%
No SCI, Replicate 3	No SCI	48,402,602	43,255,174	89.37%	4,415,622	9.12%
No SCI, Replicate 4	No SCI	41,481,052	37,178,297	89.63%	3,677,565	8.87%
Contusion SCI, Replicate 1	CONT SCI	53,820,128	48,151,362	89.47%	4,869,590	9.05%
Contusion SCI, Replicate 2	CONT SCI	43,710,401	39,145,156	89.56%	3,916,247	8.96%
Contusion SCI, Replicate 3	CONT SCI	45,022,848	40,431,294	89.80%	3,959,183	8.79%
Contusion SCI, Replicate 4	CONT SCI	43,823,672	38,905,291	88.78%	4,201,196	9.59%
Contusion SCI, Replicate 5	CONT SCI	52,408,086	46,977,110	89.64%	4,670,237	8.91%
Contusion SCI, Replicate 6	CONT SCI	46,302,615	41,341,860	89.29%	4,288,060	9.26%
Contusion SCI + SWIM, Replicate 1	CONT SCI + SWIM	36,127,931	32,173,451	89.05%	3,332,163	9.22%
Contusion SCI + SWIM, Replicate 2	CONT SCI + SWIM	22,839,371	20,305,455	88.91%	2,142,968	9.38%
Contusion SCI + SWIM, Replicate 3	CONT SCI + SWIM	28,375,965	25,194,580	88.79%	2,725,712	9.61%
Contusion SCI + SWIM, Replicate 4	CONT SCI + SWIM	42,055,999	37,629,717	89.48%	3,803,462	9.04%
Contusion SCI + SWIM, Replicate 5	CONT SCI + SWIM	38,057,288	34,067,887	89.52%	3,434,860	9.03%
Contusion SCI + SWW, Replicate 1	CONT SCI + SWW	40,038,442	35,829,624	89.49%	3,570,182	8.92%
Contusion SCI + SWW, Replicate 2	CONT SCI + SWW	42,440,037	37,813,748	89.10%	3,856,836	9.09%
Contusion SCI + SWW, Replicate 3	CONT SCI + SWW	33,579,211	30,036,726	89.45%	3,034,375	9.04%
Contusion SCI + SWW, Replicate 4	CONT SCI + SWW	28,272,049	25,189,625	89.10%	2,587,998	9.15%
Contusion SCI + SWW, Replicate 5	CONT SCI + SWW	40,461,103	36,117,639	89.27%	3,654,807	9.03%
Complete transection SCI, Replicate 1	TX SCI	28,777,268	25,745,615	89.47%	2,586,418	8.99%
Complete transection SCI, Replicate 2	TX SCI	41,441,568	36,698,047	88.55%	3,981,651	9.61%
Complete transection SCI, Replicate 3	TX SCI	54,780,102	48,712,719	88.92%	5,230,406	9.55%
Complete transection SCI, Replicate 4	TX SCI	39,452,986	34,821,163	88.26%	3,857,604	9.78%

### Quality assessment

**Fig. 3:**
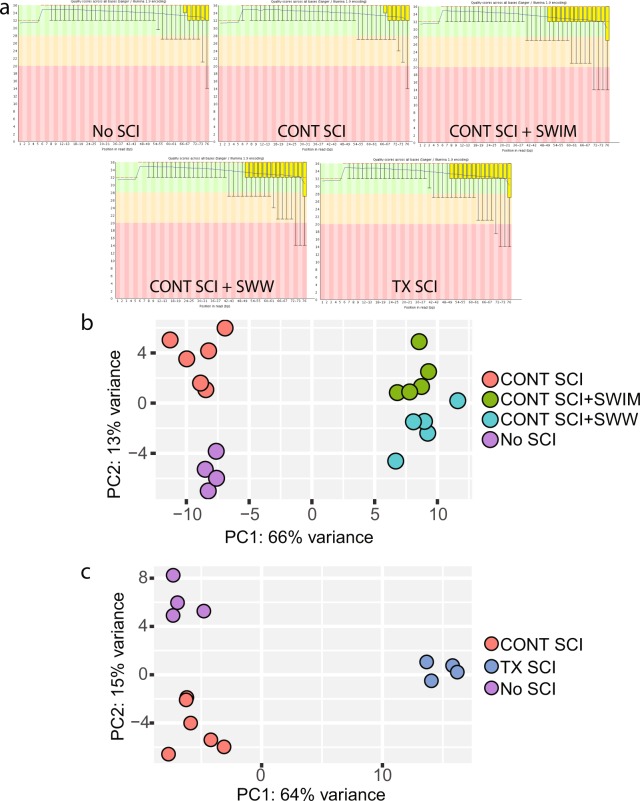
Quality control analysis. (**a**) Phred quality scores (y axis) per base (x axis) for one representative sample from each experimental group. The yellow box represents the inter-quartile range (25–75%) with the lower and upper whiskers representing the 10^th^ and 90^th^ percentiles. Scores above 28 (green) are high quality. Scores between 20 and 28 (orange) are reasonable quality, and scores below 20 (red) are poor quality. (**b**) Principal component analysis for the comparison between contusion injury and contusion injury followed by exercise training. (**c**) Principal component analysis for the comparison between the two injury models.

Raw sequencing data was input to FastQC for quality assessment. All samples were deemed of high quality. In Fig. 3a, the Phred quality score per base is displayed for a representative sample from each experimental group. With the exception of the last base in some samples, the 25^th^ percentile of quality scores is at or above a Phred score of 30, reflecting 99.9 percent accuracy in base calling. When scores at the 25^th^ percentile drop below this point on the last base, they remain in the reasonable quality category. A gradual drop in quality toward the end of the sequenced read is inherent to Illumina’s approach to sequencing by synthesis^37^.

### Gene expression variation of biological replicates

PCA was performed to assess the within- and between-group variation of the samples. The samples were separated into two distinct comparison sets, one appropriate for an examination of the effect of exercise on SCI and one appropriate for an examination of injury severity. Figure 3b displays the PCA plot for samples comparing CONT SCI alone with CONT SCI + SWIM/SWW. Samples within each experimental group cluster together, and all experimental groups lie in different regions of the graph. Sixty-six percent of the variance is explained by the separation of CONT SCI + SWIM/SWW samples and No SCI/CONT SCI samples. Figure 3c displays the PCA plot for samples with different types of injury. Once again, samples within each experimental group cluster together, and all experimental groups are located in different regions of the graph. Sixty-four percent of the variance is explained by the distinction between TX SCI samples, the more severe SCI, and No SCI/CONT SCI samples.

**Fig. 4:**
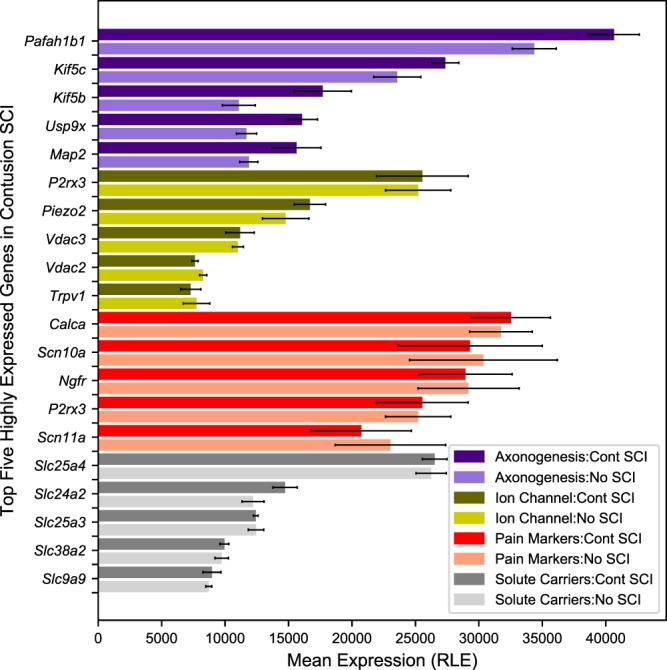
Gene expression in functional categories relevant to likely mechanisms of sensory neuron sprouting and sensitization. The five genes with highest mean expression across the six contusion injured (CONT SCI) samples are displayed for each category. Mean expression for No SCI is included as a comparison. Read counts are normalized using DESeq2’s relative log expression (RLE).

**Fig. 5:**
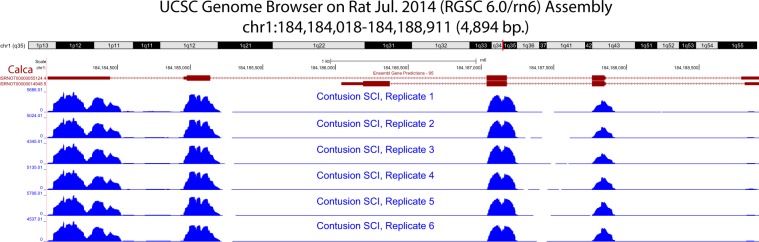
UCSC Genome Browser gene expression tracks. Custom tracks displaying *Calca (calcitonin gene related polypeptide (CGRP) alpha)* expression across the six contusion spinal cord injury (CONT SCI) samples. Expression appears consistent across the samples.

Gene expression was confirmed in SCI samples for genes relevant to sensory functions of particular interest. Genes associated with ion channels, solute carriers, and axonogenesis were selected from Ensembl (version 93) and Rat Genome Database gene function descriptions for rat^30^. Genes identified as pain markers were selected from the Pain Research Forum^38^. In Fig. 4, mean expression for the CONT SCI samples is displayed for the highest expressed genes in each functional category. Mean expression for No SCI is included as a comparison. UCSC Genome Browser tracks for the CONT SCI samples positioned at *Calca (CGRPα)* are shown in Fig. 5. Gene expression appears to be consistent for both the transcript variants at all the locations across the six CONT SCI samples, suggesting strong intra-group consistency.

### Potential batch effects

The period between injury and tissue collection varied from 8.5 to 13.5 weeks, depending on experimental condition. This was intentional and resulted from an attempt to balance the requirements of our experimental design and the well-being of the animals. The animals undergoing a complete spinal cord transection required extensive care to ensure their well-being. Given that physiological measures are known to stabilize by four weeks after complete transection^39^, it was deemed acceptable to collect tissue at 8.5 weeks, over 4 weeks beyond this “stabilized” time point. The exercised animals required an initial period of time for introduction to the exercise facility followed by 10 weeks of exercise training, resulting in the 13.5 weeks time point for tissue collection. Studies of spinal cord contusion injury indicate the transition from a sub-acute to chronic stage occurs at 4 weeks post-injury^40^. Consequently, the likelihood of batch effects is low. This is lowered further because tissue was collected by the same individual using the same methods.

## ISA-Tab metadata file

Download metadata file

## Data Availability

All analyses were performed using open sources software tools. Raw sequencing files were downloaded from Illumina BaseSpace using the Illumina Python Run Downloader^41^. Individual samples were initially divided across four lanes for sequencing, and these files were concatenated into one single end Fastq file using the UNIX cat command. cat <*FN1*>.fastq <*FN2*>.fastq <*FN3*>.fastq <*FN4*>.fastq > <*COND_REP*>.fastq The concatenated sequences were input to FastQC v.0.10.1^27^ for quality control analysis using default parameters. fastqc <*COND_REP*>.fastq -o <FASTQC_DIRECTORY> Fastq files were input to Star 2.6^28^ for alignment specifying BAM file format sorted by coordinate and requesting unmapped read files. STAR–runMode alignReads–outSAMtype BAM SortedByCoordinate –outSAMstrandField intronMotif–outReadsUnmapped Fastx–readFilesIn <*COND_REP*>.fastq.gz –outFileNamePrefix <*COND_REP*>–runThreadN 16–genomeDir Rnor_6.0 –readFilesCommand zcat Read counts for each sample were extracted using HTSeq. 0.10.0^29^. The *reverse* option was used to indicate strand orientation. Illumina’s TruSeq Stranded mRNA protocol was used to sequence the data and produces libraries where the first read is on the opposite strand to the RNA molecule. htseq-count -f bam–stranded=reverse–mode=intersection-nonempty -r name <*COND_REP*>/Aligned.sortedByCoord.out.bam Rattus_norvegicus.Rnor_6.0.93_PARSED.gtf ><*COND_REP*>/gene_counts_Reversed.htseq The raw counts were normalized using DESeq2’s^31,32^ default procedure, relative log expression (RLE), using the *estimateSizeFactors* function. Detailed instructions can be found on the Bioconductor website for DESeq2^42^.
